# Neuron-Specific Enolase as a Biomarker for Selected Neurological and Psychiatric Disorders—A Systematic Review of the Literature

**DOI:** 10.3390/medicina61101831

**Published:** 2025-10-13

**Authors:** Alicja Sierakowska, Ewa Niewiadomska, Sebastian Łabuda, Anna Bieniasiewicz, Mateusz Roszak, Beata Łabuz-Roszak

**Affiliations:** 1 Institute of Medical Sciences, University of Opole, 45-052 Opole, Poland; mateuszroszakmail@gmail.com; 2 Department of Psychiatry, St. Jadwiga Regional Specialized Hospital, 45-221 Opole, Poland; seblab@poczta.onet.pl; 3 Department of Epidemiology and Biostatistics, Faculty of Public Health in Bytom, Medical University of Silesia, 40-055 Katowice, Poland; eniewiadomska@sum.edu.pl; 4 Department of Neurology, St. Jadwiga Regional Specialized Hospital, Institute of Medical Sciences, University of Opole, 45-052 Opole, Poland; anna.bieniasiewicz@gmail.com (A.B.); beata.labuzroszak@uni.opole.pl (B.Ł.-R.)

**Keywords:** neuron-specific enolase, neuroscience, schizophrenia, ischemic stroke, psychiatric disorders, biomarker variability

## Abstract

*Background and Objectives*: Neuron-specific enolase (NSE) is an isoenzyme of enolase, of which the γ isoform is expressed in nerve cells. The activity of NSE occurs during late neuronal differentiation, which determines the specificity of the enzyme for neurodevelopmental cells. The activity of NSE is also observed in processes associated with neuronal damage. The aim of this study was to present the state of the art related to the knowledge, advances, and possible developmental directions in terms of the use of NSE as a biomarker in the diagnosis of selected neurological and mental disorders (NDs, MDs), with particular emphasis on ischemic stroke (IS) and psychotic disorders (PSDs). *Materials and Methods*: A literature review was performed using the PubMed, Embase, and Scopus databases. Keywords such as “neuron-specific enolase”, “neuron-specific enolase in schizophrenia”, “neuron-specific enolase in ischemic stroke”, “neuron-specific enolase in psychiatric disorders”, and “neuron-specific enolase in neurological diseases” were used during the literature search. A total of 11,350 items were found. However, 188 papers were finally selected after applying the filters (“clinical trial”, “meta-analysis”, “randomized control trial”, and “systematic review”). *Results*: The literature was analyzed and 67 items relevant to the subject of this study were selected. This article points out the differences in NSE levels in different clinical groups, such as patients after an incident of hypoxic/ischemic encephalopathy (HIE), neuroinfection, or particular inflammatory processes in the nervous system region, as well as central nervous system (CNS) injury, selected MD, neurodegenerative disorders (NGDs), headaches, or epilepsy (EP). *Conclusions*: In the future, they may serve to support further work on the use of enolase as a potential biomarker of the described diseases.

## 1. Introduction

Neuron-specific enolase (NSE) is an isoenzyme of enolase that plays a vital role in the glycolytic pathway, in which glucose is converted to pyruvate with the simultaneous production of adenosine triphosphate (ATP), nicotinamide adenine dinucleotide, and hydrogen (NADH) molecules. The aim of the process is to produce energy that is crucial for cellular metabolism. In vertebrates, three isoforms of enolase are identified: α-enolase (commonly found in cells), β-enolase (specific to muscle cells) and γ-enolase (typical of neurons). The expression of NSE occurs during late neuronal differentiation, which determines the specificity of the enzyme for neurodevelopmental [1], paraneuronal, and neuroendocrine cells [2]. In their study on the cellular distribution of enolase isoenzymes in the adult brain, one group found that NSE showed expression in neurons. However, it was not reported in glial cells [3,4]. Furthermore, NSE has applications in the diagnosis of neurodegenerative mechanisms and some neurological diseases. Abnormal levels of the enzyme indicate a disease and also show deviations in neurodevelopmental and neuroprotective processes [5].

NSE has also been used to quantitatively measure the extent of brain damage. It is primarily used for diagnostic and prognostic purposes in patients with intracerebral hemorrhage, coma after cardiopulmonary resuscitation (CPR) due to sudden cardiac arrest (SCA), or after a seizure episode [1]. Increased serum NSE levels may correlate with the occurrence of some cancers, including melanomas, seminomas, renal cell carcinomas, Merkel cell tumors, carcinoid tumors, germinomas, teratomas, pheochromocytomas, and neuroendocrine tumors (NETs) [6,7,8,9,10]. Currently, NSE is the most reliable biomarker for the diagnosis and treatment evaluation of small cell lung cancer (SCLC). The concentration of NSE directly correlates with tumor mass, the number and extent of metastases, or clinical response to treatment [11,12]. Studies have evaluated the usefulness of NSE as a biomarker in the diagnosis of conditions such as Guillain–Barré syndrome or Creutzfeldt–Jakob disease [13,14].

This paper presents the state of the art related to the use of NSE in the diagnosis of selected neurological and mental diseases (NDs, MDs), with particular emphasis on ischemic stroke (IS) and psychotic disorders (PSDs). Given the need for reliable clinical biomarkers in both NDs and MDs, the use of available knowledge on NSE, as well as the assessment of enzyme levels, while not solving the lack of clinical determinants, can be helpful for multimodal clinical assessment.

Despite advances and continuous development in imaging and laboratory diagnostics, there is still a lack of specific biomarkers that would enable earlier diagnosis of neurological and psychiatric diseases. Currently, diagnoses, especially of mental illnesses, are made primarily on the basis of clinical assessment, which carries the risk of diagnostic bias due to the subjectivity of the examiner. This often leads to delays in the initiation of adequate treatment. Biomarkers could contribute to increased diagnostic accuracy and, at the same time, earlier initiation of the required therapy, often giving a more favorable prognosis for the further development of the disease. The search for markers such as NSE, which reflects neuronal damage as well as neurodegenerative and neuroprotective processes, seems particularly promising.

## 2. Materials, Methods, and Results

Introduction: Data extraction was performed independently by two reviewers. Discrepancies were resolved through discussion, and no automation tools were used in the process. In addition to outcomes related to neuron-specific enolase (NSE) levels, we extracted information on study design, sample size, participant characteristics (e.g., age, sex, clinical diagnosis), country, and funding sources when available. Given the heterogeneity of included studies, no single quantitative effect measure was used.

Materials and Methods: Results were synthesized narratively, and summary statistics reported by the original studies (e.g., means, standard deviations, or *p*-values) were extracted when available. A narrative synthesis approach was adopted due to the diversity of study designs, populations, and outcome measures. Findings were organized thematically according to neurological and psychiatric conditions. No meta-analysis was conducted; therefore, no statistical heterogeneity, subgroup analyses, or sensitivity analyses were performed. A systematic literature review was performed using the PubMed, Embase, and Scopus databases. Formal tools to assess the risk of bias and the certainty of evidence (e.g., GRADE) were not applied, given the narrative scope of this review. This review was not registered in PROSPERO or any other database, and no prior review protocol was prepared. The keywords “neuron-specific enolase” OR “neuron-specific enolase in schizophrenia” OR “neuron-specific enolase in ischemic stroke” OR “neuron-specific enolase in psychiatric disorders” OR “neuron-specific enolase in neurological diseases” were used during the literature search (n = 11,350 results). 

Results: Next, 1800 papers were rejected due to duplication in other databases. After applying filters (“clinical trial,” “meta-analysis,” “randomized control trial”, and “systematic review”), rejecting papers where only an abstract was available, or where the paper was an expert opinion or conference article, 188 publications were selected. In this study, the main goal was to analyze the dissimilarity in NSE in severe mental illnesses (such as schizophrenia or psychosis and bipolar affective disorder) as well as the most common neurological diseases (ischemic stroke, epilepsy, headaches, neurodegenerative or neuroinflammatory conditions); therefore, we primarily included publications that meet the above criteria. In total, 110 items were considered relevant in terms of the issues addressed, 25 of which were written in a language other than English, while 23 described the results of studies that had not been completed. In the end, 67 items were considered.

**Figure 1:**
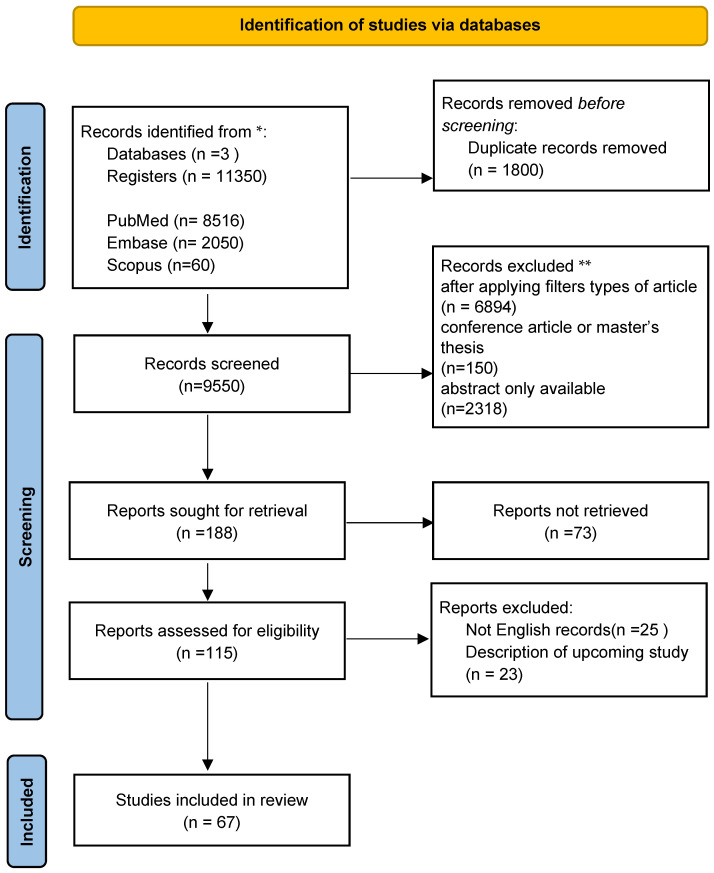
The source selection presented as the PRISMA flow diagram (Haddaway et al., 2022 [16]). * Consider, if feasible to do so, reporting the number of records identified from each database or register searched (rather than the total number across all databases/registers). ** If automation tools were used, indicate how many records were excluded by a human and how many were excluded by automation tools.

The detailed process of source selection is presented in the form of a graph—Figure 1.

Discussion: Reporting bias and publication bias were not formally assessed. The certainty of the evidence was not formally evaluated.

The authors declare that this review has been prepared in accordance with the PRISMA (Preferred Reporting Items for Systematic Reviews and Meta-Analyses) guidelines [15] (Supplementary Materials). The PRISMA checklist and flow diagram were applied to ensure the methodological transparency, accuracy, and reproducibility of the findings.The authors conducted a systematic literature search in PubMed, Embase, and Scopus databases from 1982 to 2025. The search combined the controlled terms “neuron-specific enolase” OR “neuron-specific enolase in schizophrenia” OR “neuron-specific enolase in ischemic stroke” OR “neuron-specific enolase in psychiatric disorders” OR “neuron-specific enolase in neurological diseases”, and free keywords related to “neuron-specific enolase”, “biomarker in mental disorders”, or “biomarker in neurological disorders.” Initially, no language restrictions were applied, but only studies published in English were included in the analysis. 

Conclusions: Original scientific articles, randomized clinical trials, observational studies, and systematic reviews were included in this study. The exclusion criteria were as follows: works in the form of a conference abstracts without the full text, editorial articles, case reports, and works lacking relevant data on results. Two reviewers independently selected titles and abstracts, and then analyzed the full texts. Discrepancies between the various search processes and the qualification of works for this study were resolved by consensus.

### 2.1. NSE Kinetics

The half-life of the enzyme is estimated to be about 30 h [17]. However, in individual body fluids, the dynamics vary. For cerebrospinal fluid (CSF), it is typically between 2 and 4 h. The short half-life in CSF is important in the context of monitoring pathological conditions such as brain damage, due to the possibility of predicting changes in the patient’s clinical picture. In blood serum, the half-life oscillates from about 18 to 24 h. Due to the longer presence of the enzyme in the blood, it gains the following applications: in the diagnosis of neuroendocrine tumors or further monitoring and clinical prognosis among patients after central nervous system (CNS) injury, SCA, and other kinds of HIE. In the case of urine, the half-life of NSE is not clearly defined, as the evaluation of concentration values has not yet been applied in clinical practice. The change in NSE concentration values depends on factors such as renal function and the patient’s overall clinical condition [1]. Currently, no studies have been published that can clearly determine the half-life directly in specific brain areas. Moreover, it should be noted that there are currently no standard limit values for NSE levels in individual body fluids (cerebrospinal fluid, blood serum, urine). This is most likely the reason for the numerous variations and discrepancies in the studies listed below.

### 2.2. Hypoxic/Ischemic Processes in the Central Nervous System

Hypoxia and cerebral ischemia are usually caused by (SCA) and stroke (S). According to the literature, serum NSE levels and calcium-binding protein (S100B) measured in urine are the biomarkers of greatest clinical relevance indicating hypoxic/ischemic encephalopathy (HIE) [18]. The pathomechanism of NSE release from the cell is related to the collapse of the cell membrane due to its damage [1]. Many studies have suggested that measurements of serum levels of NSE obtained from neonates who presented with HIE during the perinatal period may be helpful in assessing the prognosis of survival [19]. Some reports have demonstrated an association between NSE levels and the volume of the brain area involved in ischemic stroke (IS). However, due to the absence of clinical trials, this marker is not currently considered conclusive [20]. Some researchers reported on the possibility of predicting a good functional outcome at 12–14 days after the onset of acute IS if the severity of neurological symptoms was below a score of 15 (National Institutes of Health Stroke Scale; NIHSS) at onset and the NSE concentration in the cerebrospinal fluid (CSF) was <2 ng/mL in first 24 h [21].

There are ongoing studies on the use of NSE in assessing the severity of postoperative cognitive dysfunction (POCD). In one randomized controlled trial (RCT) available in the literature, patients undergoing pre-urethral resection of the prostate (TURP), covered during surgery by general anesthesia with propofol, were assessed for cognitive function before surgery and on the first, second, and ninth days after surgery. The results indicated higher levels of NSE in patients with POCD compared to a group of participants who did not have these disorders, as well as a control group [22].

NSE has also been used to predict neurological status in patients who presented with (SCA). Sandroni and D’Arrigo showed that 72 h after the return of spontaneous circulation (ROSC), somatosensory evoked potentials (SSEP), electroencephalography (EEG), brain imaging studies, and the measurement of serum NSE levels were crucial, alongside the clinical evaluation of the patient [23]. Currently, the level of NSE decreases after 24 h in patients with a good prognosis, while the NSE concentration increases in patients with an unfavorable prognosis. The highest levels are found between 48 and 96 h after ROSC [24,25].

A recent study has indicated an association between the prognostic value of NSE depending on age and the extent of CNS damage. Wihersaari et al. demonstrated that the greatest statistical significance and utility of NSE was observed in younger patients with the longest time to ROSC after SCA [26]. Nevertheless, the 2021 European Resuscitation Council (ERC) guidelines focused on an unfavorable prognosis in patients after SCA and considered a serum NSE level >60 ng/mL the cutoff point, and also indicated the need for multimodal prognostication [27]. An important aspect of using NSE to predict unfavorable long-term outcomes in patients after SCA is defining the number of determinations and when they should be performed. According to Wiberg et al., three measurements of NSE levels should be performed after ROSC, i.e., at 24, 48, and 72 h [28]. 

Similar studies were also conducted on a group of Wistar rats. Sixteen males were included in the experiment, half of which (the study group—HIE) underwent occlusion of the right common carotid artery, after which they were placed in a hypoxic chamber and subjected to reperfusion after 60 min. NSE concentrations were measured and the clinical condition of the rats was assessed for 24 h. The results of the preliminary neurological examination indicated the presence of hemiparesis in all individuals from the HIE group. In addition, NSE concentrations in this group were significantly higher than those in the control group [29].

### 2.3. Neuroinfections and Selected Inflammatory Processes

Both clinically silent and symptomatic Sars-CoV-2 viral infections are significant in terms of changes in NSE levels. A study conducted in patients from northeast Brazil showed elevated NSE levels in patients with severe and mild symptoms of COVID-19 infection. However, no correlation was observed between the presence or severity of neurological symptoms resulting from the Sars-CoV-2 virus and NSE levels. Furthermore, the follow-up study demonstrated a decrease in NSE levels over time (21 days after COVID-19 infection), which suggested only a mild level of neural injury [30].

NSE has proven to be a promising marker from the perspective of diagnosing acute bacterial cerebrospinal meningitis (ABM). Researchers attempted to determine the temporal profile of NSE, as well as looking for correlations between the course of the disease and prognosis of ABM, and the substance’s concentration values. Determinations of serum NSE levels were performed three times each day of the patient’s stay in the unit. The values obtained were significantly higher among patients with a diagnosis of ABM compared to the healthy population; moreover, an increase was observed within the first 3 days of patient admission. On day 5–6, the recorded levels of the enzyme did not exceed those observed at the beginning of hospitalization [31].

Septic encephalopathy (SE) ranks as the second most common manifestation of brain infection [32]. The potential role of NSE in determining the extent of neuronal damage in SE remains questionable, due to the dependence of enzyme levels on hemolysis, which also takes place during sepsis (which is why the authors suggest determining NSE only in non-hemolytic plasma or serum samples). Available analyses report that each twofold increase in concentration values from the level determined at admission was associated with a 7% increased risk of death within 30 days and a 5% increased risk of delirium [33]. Among newborns, NSE levels assessed in cord blood were statistically higher for patients with SE, and were moreover associated with a higher likelihood of impaired neural development over the next 6 months of life [34].

NSE levels were also assessed in patients with various forms of tick-borne encephalitis (TBE). The study findings indicated higher NSE levels in patients with TBE compared to those with meningitis. In addition, the levels of NSE were different between the clinical and control groups. Additionally, NSE concentrations increased after 14 days of treatment compared to the results from the samples collected before treatment [35]. The evaluation of NSE levels was also used in assessing nerve tissue damage in patients with post-herpetic neuralgia (PHN). Zhong et al. conducted a study on groups of patients with mild and severe PHN based on a 100-point Likert scale, which corresponded to the severity of the neuralgia. The NSE levels in patients with PHN were higher compared to healthy individuals. In addition, a positive correlation was found between the severity of neuralgia and NSE levels [36]. NSE concentrations were also assessed in patients with pulmonary tuberculosis (TB). In their retrospective analysis, Nam SJ et al. determined the predictive value of NSE based on a group of patients with focal segmental or extensive TB (based on chest X-ray) and a control group. NSE was determined before and after treatment. Significantly higher NSE levels were observed in the participants from the study group compared to healthy controls. An increase in NSE levels was observed in patients with extensive TB compared to focal segmental TB [37].

### 2.4. Traumatic Brain Injury

Traumatic brain injury (TBI) causes disability and is associated with the most widespread effects. It involves emotional and behavioral disorders and permanent physical impairment [38]. Depending on the severity of injury, TBI is divided into mild (a consequence of concussion, which leads to the disruption of the axonal structure without further consequences associated with the functioning of the axonal structure; and a 14–15 score on the Glasgow Coma Scale [GCS]); moderate (most often due to hemorrhagic brain contusion due to direct damage to neurons and impaired conduction of nerve impulses in the brain; 9–13 on the GCS); and severe (most often due to diffuse axonal damage related to the damage to axons in the region of the corpus callosum and brainstem; 3–8 on the GCS) [38,39].

Research is currently underway to find biomarkers that could be useful in predicting the extent of injury and the patient’s ultimate functional status. Previous reports have confirmed the usefulness of measuring several times and, at the same time, assessing the dynamics of NSE levels over elapsed time. An example of this is a work in which NSE determinations were made in patients who had suffered a TBI (taking into account the division into mild and severe injuries, depending on the GCS score obtained) at the time of admission to the hospital, after 6, 12, 24, and 48 h, and 2–7 days of hospitalization. In addition to statistically significant positive correlations between TBI severity and NSE concentration values, there was an upward trend in biomarker values in individual measurements with mild injuries, and an intense increase followed by a periodic decrease in NSE among patients with a history of severe TBI [40]. Other reports [41] evaluated the dynamics of enzyme changes (at 1, 2, 3, 4, and 5 days after injury) in patients after TBI by dividing them into groups, including participants who experienced hypoxia and patients with normal tissue oxygenation resulting from injury. Statistically significant differences were observed, indicating higher levels of NSE in those with hypoxemic injury, as well as a persistent downward trend in scores with time. Reports are also available showing an unexplained increase in NSE levels 3 days after brain injury [42,43,44]. According to some reports, a decrease in NSE concentrations was observed in TBI survivors within 48 of injury [45,46]. In turn, some clinical studies indicated a more rapid decrease in serum levels of NSE in post-TBI subjects compared to healthy individuals [46]. Data are conclusive as to the half-life of NSE, which is 48–72 h [40,45,46,47,48]. In an experiment aimed at determining a possible link between the severity of consciousness disorders in patients after severe brain injury and NSE, higher levels of this marker were found in the patients whose GCS score was 3, which corresponded to clinical brain death compared to those with a GCS score of 4–8 [49].

As regards the prevalence of head injuries, it seems important to also measure NSE in individuals who present with repetitive subconcussive head impacts (RSHIs). Studies in this respect have been mostly conducted on boxers, American football players, and soccer players [49]. Each of the studies showed an increase in NSE levels immediately after matches or boxing bouts. Furthermore, the levels were significantly higher compared to the laboratory norm (soccer games causing a 1.1–2-fold increase, American football a 1.9-fold increase, and boxing a 1.6–2.5-fold increase in NSE) [50].

In order to broaden the clinical perspective, the authors decided to also cite a study conducted on animal cells. As in the above studies, the aim was to assess changes in NSE concentration in blood serum after TBI in a group of Wistar rats. Therefore, a group of 65 males underwent TBI, and then after 1, 6, 12, 24, and 48 h, blood samples were taken from them and NSE levels were measured. Time-dependent changes in NSE concentration were demonstrated. The highest values were recorded 6 h after brain injury, and a correlation was suggested between the level of neuronal damage resulting from cerebral cortex contusion and an increase in NSE levels [51].

### 2.5. Mental Disorders

Mental disorders (MDs) are diagnosed based on the patient’s clinical observation. Currently, there are no proven biomarkers, imaging studies, or other predictive factors that could clearly confirm or exclude a disorder. Many studies have been conducted to search for an adequate determinant that could decrease human error often associated with misdiagnosis [52]. Of a large number of biomarkers under investigation, NSE could be identified as a substance that has recently been widely investigated.

In 2012, Streitbürger et al. performed imaging and histological and genetic analysis of the brains of subjects without clinical factors that could influence a change in NSE levels. Women showed a negative correlation between NSE expression and gray matter density in both the amygdala and most of the anterior hippocampus [53].

Many studies have focused on the correlations between NSE concentrations and the occurrence of psychotic disorders (PSDs). A pioneering post mortem study conducted before 1987, on a group of 9 patients diagnosed with schizophrenia (SZ) and 15 healthy volunteers (HC), indicated that NSE levels were 70% higher in the sensory cortex and 70% lower in the thalamus in patients with SZ compared to the controls. In addition, a 15–20% increase in NSE levels was also observed in the temporal cortex. However, a similar percentage decrease was reported in limbic system structures in patients from the study group [54].

Over the following years, various reports have indicated a significant difference in NSE levels between the group of patients with the first episode of schizophrenia (FES) (who presented with higher enolase levels) and the controls [55]. The extent of remission and the effectiveness of drug treatment also play a significant role. In their study, Liu et al. compared NSE concentrations in patients with untreated first-episode schizophrenia (uFES) and subjects with medicated chronic schizophrenia (CSZ) and the controls. Mean NSE concentrations were lower in the CSZ group compared to the uFES group and the HC [56].

Different findings were presented by Andreou et al. Their study involved adolescent and adult patients with SZ, bipolar spectrum disorder (BD), and healthy controls (HC). NSE levels were significantly lower in adults and adolescents from the study group compared to the HC. Furthermore, both subgroups showed a negative correlation between NSE levels and symptom severity. The results were significant after controlling for total gray matter volume. The authors postulated that the obtained data reflected abnormal neuronal maturation in patients with MD, which supports the thesis regarding the neurodevelopmental mechanism of severe MD [57]. Assessments of serum NSE can also be used in other aspects of research on SZ. In their study, Taler et al. analyzed the relationship between the permeability of the blood–brain barrier (BBB) and the occurrence of PSD in patients with a 22q11.2 mutation (which predisposes to psychiatric disorders) by measuring NSE levels (also measured were levels of S100B protein, elevated values of which, like NSE, indicate damage to neural tissue). They found an increase in BBB permeability in patients with the mutation and confirmed previous theses regarding a new neuronal pathway predisposing to psychosis with the 22q11.2 genetic defect [58].

### 2.6. Neurodegenerative Diseases

First, it is worth mentioning the described non-glycolytic function of NSE, which may play a role in the pathophysiology of neurological diseases, particularly neurodegenerative diseases. The C-terminal fragment of γ-enolase has been shown to have a neurotrophic effect, which promotes the survival of individual neurons and nerve processes by activating the PI3K/Akt and MAPK/ERK pathways (responsible for the indicated processes) [59]. The described processes have also been confirmed in more recent reports, on the basis of which conclusions have also been drawn regarding the involvement of NSE in neuroprotective and regenerative mechanisms in the CNS [60].

Research is conducted on the use of NSE levels in the diagnosis of neurodegenerative diseases (NGDs). In their study on NSE levels in Alzheimer’s Disease (AD), Schmidt et al. assessed the CSF from 32 patients diagnosed with AD and 32 controls. The mean NSE levels were statistically significantly higher in the study group compared to the results from the control group. Furthermore, in combination with Tau protein, whose presence is associated with the pathophysiology of AD, elevated NSE levels (above the upper limit of normal range = 15.80 ng/mL) were crucial in diagnosing AD [61]. Although the diagnosis of Parkinson’s disease (PD) is made on the basis of fulfilled clinical symptoms, additional evaluation of NSE concentration values seems reasonable. In their study on 58 patients with PD and 28 controls, Papuć and Rejdak determined the relationship between the presence of particular molecules in the CSF and the occurrence and severity of PD. The concentration of NSE in the CSF was significantly higher in the study group than in the controls. However, no correlation was found between NSE levels and the severity and duration of PD. NSE levels in CSF provided relatively high discrimination (AUC 0.775) between PD and healthy controls, with a sensitivity of 78.6% and specificity of 74.1% at a cutoff value of 51.56 ng/mL (*p* < 0.001) [62].

The meta-analyses that focused on the comparison of NSE concentrations in conditions such as AD, PD, Parkinson’s disease with dementia (PDD), dementia with Lewy bodies (DLB), or multiple system atrophy (MSA) showed no significant differences. However, the analysis of the subgroups (PDD and DLB) indicated increased NSE levels in the CSF. Therefore, this biomarker can be useful in assessing the severity of neurodegenerative processes in AD and PDD/DLB [42]. In another study, which aimed to compare NSE levels in patients with multiple sclerosis (MS), the relationship between clinical disability and the levels of NSE was assessed at the beginning and the end of the follow-up. However, no significant relationships were found [55].

Similar observations were made when comparing NSE levels in patients in an inactive primary progressive multiple sclerosis (PPMS) phase II clinical trial treated with hydroxychloroquine formulations at a dose of 200 milligrams twice daily for a period of 18 months. The study found no statistically significant differences [35].

Tsukahara et al. [63] evaluated NSE levels in the CSF in patients with amyotrophic lateral sclerosis (ALS) and cervical spondylotic myelopathy (CSM). Their study included 45 patients with ALS, 23 with CSM, 10 with PD, and 28 controls. The study group showed significantly higher NSE levels compared to the controls. In addition, an increase in NSE levels was observed in patients with ALS compared to those with CSM [36].

### 2.7. Headache

Given the many etiopathogenetic theories of migraine (MG), studies have been conducted on specific clinical parameters in patients with MG. Yilmaz found lower serum concentrations of NSE and higher S100B levels in patients with MG compared to the control group with no history of headache. Furthermore, a moderate positive linear correlation was reported between the molecules [37]. However, different results were presented by Gönen et al., who compared NSE levels in several groups (patients with episodic MG with aura, patients with episodic MG without aura, patients with chronic MG and healthy volunteers).

The results of the measurement of NSE levels in patients with MG were significantly higher compared to the healthy volunteers. Furthermore, the mean NSE concentrations of the participants diagnosed with chronic MG exceeded NSE levels in patients with episodic MG [64]. Nonetheless, it is important to keep in mind the small number of participants involved in each study, which most likely explains the discrepancy in the results obtained. No statistically significant differences were found in NSE levels in different subgroups based on randomized clinical trials conducted in patients with cluster headaches (bout, remission, and chronic cluster headache) [65].

### 2.8. Epilepsy

Epilepsy (EP) affects approximately 50 million people worldwide. Predicting the clinical course and adequately treating it are extremely costly, which is why numerous biomarkers have found their way into both the diagnosis and evaluation of the disease’s treatment efficacy [66]. NSE is one of the predictors described. Although the first reports indicating significant differences in the concentrations of substances in people with a diagnosis of epilepsy compared to the general population date back to the end of the last century, the topic has still not been thoroughly investigated. 

Available meta-analyses indicate a significant increase in NSE levels in both cerebrospinal fluid and blood serum among a population of children with a diagnosis of EP compared to a HC. In addition, a positive correlation was found between NSE concentrations and the severity of the disease [67]. In one report, the authors detailed groups of patients with complex partial seizures and secondary generalized tonic–clonic seizures. They measured NSE levels at 1, 3, 6 and 24 h after the onset of an epileptic seizure. At individual measurements, a statistically significant difference was shown only between the first and last measurements. No significant differences were observed between the given clinical groups; moreover, 1 h after the onset of a seizure, only 33% of the participants had NSE concentration values above the upper limit of the laboratory norm [68]. Status epilepticus (SE) is defined as a prolonged epileptic seizure. Prompt recognition of SE allows for the initiation of treatment, consequently preventing the development of often severe neurological sequelae [69]. 

In the cited study, serum NSE levels were determined in SE patients and a control group. The results indicated significantly higher concentrations of the biomarker among those with SE [15]. Similar results were obtained in other reports [21], where serum NSE levels were compared between patients with convulsive status epilepticus (CSE) and a HC. Among the clinical group population, they reported statistically significantly higher values of the enzyme compared to participants in the HC. Increasingly, researchers are attempting to assess the use of pharmacotherapy using known biomarkers. An example of which is the work aimed at determining the therapeutic efficacy of sodium valproate in monotherapy and in combination with levetiracetam in a pediatric population using serum determinations of NSE levels, among others. On the basis of the differences in the values of NSE concentrations between the groups before and after the implementation of drug treatment (greater dynamics of changes in the values of NSE concentrations in the case of patients treated with two preparations), a conclusion was drawn as to the advantage of combination therapy with sodium valproate and levetiracetam compared to monotherapy [70,71].

### 2.9. Limitations

One of the goals of this publication was to compile and present the available knowledge on the role of neuro-specific enolase in the diagnosis, treatment, and prognosis of the course and sequelae of psychiatric and neurological diseases. For this reason, this publication includes articles labeled “clinical trial,” “meta-analysis,” “randomized control trial”, and “systematic review.” The result was the inclusion of a large number of different types of publications, which made the analysis much more difficult. Attention was paid mainly to hypoxic/ischemic processes, brain injury, and psychiatric disorders, while aspects such as neuroinfections, neurodegenerative diseases, epilepsy, and headache were presented in less detail. It should also be noted that the groups of participants—who were included in the mentioned studies—varied in size, and the researchers used different methodologies (e.g., different biological material and heterogeneous time intervals between measurements). Moreover, bearing in mind the kinetics of NSE and its bioavailability in the various body fluids in which the biomarker was measured, such different results in terms of NSE levels could be explained.

## 3. Conclusions

Recently, NSE has been analyzed extensively in terms of changes in serum levels in patients with brain damage in the form of HIE, IS, TBI, MD, and NGD. In the case of patients after neural tissue damage resulting from HIE, changes in NSE levels are already evident in the neonatal period among children with perinatal hypoxia. The authors of the work also note the relationship between the area of IS and the level of the biomarker. In addition, it has become possible to predict the functioning of post-stroke patients, taking into account the result of the NISHH sacculus and the level of NSE. The researchers also observed an association between POCD severity and higher NSE concentrations. The validity of assessing NSE levels has been shown in the prognosis of patients after SCA—a decrease in NSE levels at 24 h after the onset of ROSC, combined with the results of SSEP in EEG, indicates a successful prognosis.

Considering the variable levels of NSE in different MDs, attention is drawn to the increase in the biomarker’s concentrations in the sensory cortex and temporal cortex structures, and the decrease in the limbic system or thalamus among patients with SZ. Nevertheless, the available results of studies aimed at determining the relationship between NSE concentrations and the occurrence of SZ, BD, or the severity of symptoms remained heterogeneous. The reports presented on the increase in NSE levels support the role of the neurodevelopmental theory in the etiopathogenesis of SZ.

In inflammatory and infectious diseases (IDs), a correlation was observed between the increase in NSE levels and the presence of Sars-CoV-2 infection (both in symptomatic and asymptomatic manifestations), while no correlation was observed between the severity of infection symptoms and the values of enzyme concentrations. Determination of biomarker levels seems reasonable during ABM, where, in addition to the correlation between increased NSE concentration values and the presence of infection, higher levels of the substance were noted during the first three days of ID. Interpretation of enolase results from patients with SE has clinical relevance only if the material collected is from non-hemolytic plasma or serum samples. Among neonates, NSE levels are higher in patients with SE, and have been associated with a greater likelihood of impaired neural development. The available results indicate an association between increased NSE levels and the risk of disorder and death. Correlations between elevated enolase levels have also been observed in association with the occurrence of NSE, PHN, and TB.

Numerous papers have focused on the correlation of NSE levels with a history of TBI. In addition to statistically significant positive correlations between TBI severity and NSE concentrations, there is a characteristic initial increase and subsequent decrease in NSE, with the highest results in patients who are clinically brain-dead. An increase in biomarker concentration values was also noted in subjects exposed to repeated head trauma as a consequence of combat sports, American soccer, and soccer.

Of particular importance was the determination of NSE levels in NGDs such as AD and PD, where an increase in levels was observed in patients suffering from these conditions. Moreover, in the case of AD, in conjunction with Tau protein evaluation, an increase in NSE values above the reference value has become crucial in the diagnosis of the disease. An increase in biomarker levels has also been attributed to the presence of DLB and PDD. The results of available studies in the use of NSE in the diagnosis of MS, MG, or cluster headaches remain mixed.

In terms of EP, reports on enolase show promise. The results presented here show a significant increase in NSE levels in both CSF and serum in patients diagnosed with EP. In addition, a positive correlation was found between biomarker levels and disease severity. Similar correlations were noted among patients with SE. Based on the differences in the values of NSE concentrations between groups of patients with EP before and after the implementation of drug treatment, it became possible to draw conclusions about the advantage of combination therapy with sodium valproate and levetiracetam compared to monotherapy.

Nevertheless, in individual chapters on schizophrenia, epilepsy, and neurodegenerative diseases, the reports often present contradictory results. This was most likely due to the heterogeneity of the research methodology, the small number of participants (sometimes only a few) qualified to participate in the experiments, and differences in the selection of biological material from which the measurements for the compared studies were obtained.

Given the significant discrepancies between different studies and heterogeneous groups, further research is warranted on NSE as well as the determination of its usefulness as a biomarker in various neurological and psychiatric conditions. The use of NSE in MD seems particularly relevant. The small number of papers does not allow for a clear indication or exclusion of the clinical utility of the biomarker. Nevertheless, the study findings indicate important correlations from the perspective of planning future research and the development of clinical diagnosis. In order to expand the use of NSE, which currently remains a promising but experimental biomarker, it seems reasonable to standardize NSE measurements by selecting a specific type of body fluid in which the marker should be determined, as well as defining reference ranges for substance values or measurement techniques. The described procedure would solve the issue of methodological heterogeneity, and at the same time the often divergent results resulting from it. Moreover, it is necessary to conduct further longitudinal clinical studies on the basis of which the predictive value of the enzyme could be determined (Table 1).

Despite numerous reports on NSE, there are still gaps in research, which at the same time provide scope for future development of NSE as a potential biomarker.

These include a lack of standardization in measurement methods, heterogeneity in the study population, low clinical specificity of NSE, insufficient data on the specificity of the enzyme, and the current lack of randomized, double-blind controlled studies, or integration with modern imaging methods or artificial intelligence.

### 3.1. Study Strengths

NSE, as well as other biomarkers in a variety of neurological and psychiatric conditions, appears to be an important topic in terms of disease diagnostic development.

This paper provides a comprehensive review of a wide range of studies, integrating findings from many clinical contexts.

This paper can be used to plan future research and evaluate the development of NSE application in neurological and psychiatric diseases.

### 3.2. Weaknesses of the Study

Due to the considerable amount of available studies, this review is not able to cover all reports on the indicated topic, from where it was necessary to focus only on selected areas.

Due to the heterogeneity of the obtained material used to determine the concentration of NSE, and the often small size of the groups of participants included in this study (which is related to the continuous development of knowledge and research on enolase), it became impossible to unequivocally opine on usefulness in terms of determining NSE as a biomarker or predictive factor indicating the exact cutoff threshold of the concentration of the substance.

### 3.3. Further Research

Due to the lack of biomarkers for making a clinical diagnosis based on the results of laboratory, imaging, or functional tests, it is not uncommon for clinicians to face the problem of qualifying the symptoms presented by a patient in terms of meeting the criteria for a given psychiatric or neurological disease. Identifying individual substances would offset the risk of human error while increasing the accuracy of the diagnosis. In many cases, it would enable patients to receive earlier treatment, improving quality of life, and perhaps increase the effectiveness of the drugs used, while reducing drug resistance or the far-reaching effects of untreated disease.

Necessary further research on NSE should include a larger group of participants to resolve the often contradictory results presented. In addition, attention should be paid to the collection of the particular clinical material from which the results of the labeled enzyme are derived. The best solution would be to standardize the method of material collection (for example, the choice of blood serum) in the next experiments conducted, which would facilitate the method of obtaining a sample in almost every clinical group.

It also seems reasonable to define homogeneous reference values of NSE, which would provide an opportunity to compare the results of reports, and at the same time confront them, which would result in faster development of knowledge regarding the possibility of using NSE.

Further work should focus on determining the relationship between the severity of symptoms of individual diseases and the results of the NSE level, which would provide an opportunity to assess the severity of the disease. Moreover, clinical evaluation in terms of the improvements made from the use of individual drugs would prevent the long-term use of inappropriate preparations and their associated adverse effects.

### 3.4. Risk of Bias

This paper was written taking into account the various points of the PRISMA protocol. Databases such as PubMed, Embase, and Scopus were used, using the following keywords: “neuron-specific enolase” OR ‘neuron-specific enolase in schizophrenia’ OR ‘neuron-specific enolase in ischemic stroke’ OR ‘neuron-specific enolase in psychiatric disorders’ OR “neuron-specific enolase in neurological diseases.” We used filters such as “clinical trial,” ‘meta-analysis,’ ‘randomized control trial,’ and “systematic review.” The literature review was conducted by three authors, working independently of each other. Out of the 185 publications meeting the above criteria, 64 items were finally included in this paper, which were related to the objectives of this study, focusing on presenting the dissimilarity of NSE in severe mental illnesses (such as schizophrenia, psychosis, bipolar disorder, and affective disorders), as well as the most common neurological diseases (ischemic stroke, epilepsy, headaches, and neurodegenerative or neuroinflammatory conditions).

## Figures and Tables

**Table 1:** Changes in NSE concentration in specific diseases.

Disease	NSE Change Template	Clinical Remarks
**Ischemic stroke/Hypoxia**	↑ NSE in CSF within 24–72 h; higher NSE concentrations correlate with larger infarct size; prognosis of poor outcome with persistent values >60 ng/mL after 72 h (according to ERC guidelines);	Potential biomarker of damage and prognosis; repeated measurements necessary (24/48/72 h);
**Postoperative cognitive disorder**	↑ NSE in patients with POCD after general anesthesia;	Can be used to support early detection of postoperative complications;
**Neuroinfections (COVID-19, ABM, sepsis, TBE, PHN, tuberculosis)**	↑ NSE; ABM—the highest level in the first 3 days; Sepsis—2-fold increase correlates with a higher risk of death; TBE and PHN—correlates with severity	Different dynamics depending on the type of infection;
**Traumatic brain injury**	↑ NSE proportional to severity (GCS); peak at GCS = 3 (brain death); RSHI (boxing, football, soccer)—temporary increase after exercise;	Monitoring dynamics (0–7 days) may be helpful in assessing prognosis;
**Schizophrenia/Psychotic disorders**	Ambiguous results: ↑ NSE in the first episode psychosis; ↓ in chronic schizophrenia; correlations with symptom severity: changes in various areas of the brain (↑ in the sensory cortex/↓ decrease in the thalamus and limbic system)	No consensus
**Alzheimer disease**	↑ NSE in CSF; in combination with tau protein, an important diagnostic marker;	Supports AD diagnosis, but less specific than tau/amyloid;
**Parkinson disease**	↑ NSE in CSF; no correlation with the duration and severity of PD; sensitivity 78.6%, specificity 74.1% at a cut-off of 51.56 ng/mL	Moderate usefulness;
**Other neurodegenerative diseases (PDD, DLB, ALS, MS)**	↑ NSE in PDD/DLB and ALS; no differences in MS	A biomarker of neuronal damage, but with varying values depending on the particular disease;
**Migrane**	Results are inconsistent in terms of upward or downward trends in NSE values (especially in chronic migraine); no differences in cluster headaches	Insufficient clinical evidence
**Epilepsy/status epilepticus**	↑ NSE in CSF, correlation with disease severity; significantly higher in SE; transient increases after seizures	Possibility of supporting diagnostics and monitoring therapy (e.g., more favorable dynamics of changes in treatment with valproate + levetiracetam)

## Supplementary Materials

The following supporting information can be downloaded at: https://www.mdpi.com/article/10.3390/medicina61101831/s1.
